# Regulation of MAPKs Signaling Contributes to the Growth Inhibition of 1,7-Dihydroxy-3,4-dimethoxyxanthone on Multidrug Resistance A549/Taxol Cells

**DOI:** 10.1155/2016/2018704

**Published:** 2016-06-15

**Authors:** Jian Zuo, Hui Jiang, Yan-Hong Zhu, Ya-Qin Wang, Wen Zhang, Jia-Jie Luan

**Affiliations:** ^1^Department of Pharmacy, Yijishan Hospital, Wannan Medical College, Wuhu 241000, China; ^2^Anhui Provincial Engineering Technology Research Center of Polysaccharides Drugs, Wuhu 241000, China

## Abstract

1,7-Dihydroxy-3,4-dimethoxyxanthone (XAN) is a bioactive compound isolated from* Securidaca inappendiculata* Hassk. and validated with antiproliferative activities on a panel of cancer cell lines. This study was designed to investigate its growth inhibitory effects on multidrug resistance (MDR) non-small cell lung carcinoma (NSCLC) cell line A549/Taxol and explore the possible linkage between modulation of MAPKs and the bioactivities. Its growth inhibitory potency on the cells was estimated by MTT assay, and flow cytometric analysis was employed to investigate its potential cell cycle arrest and proapoptosis effects. Expressions of hallmark proteins were assessed by Western-Blot method. The results showed A549/Taxol cells were sensitive to XAN. XAN inhibited the proliferation of A549/Taxol cells in the time and concentration dependent manners. It acted as a potent inducer of apoptosis and cell cycle arrest in the cells. Western-Blot investigation validated the proapoptosis and cell cycle arrest activities of XAN and the potential of MDR reversion. Upregulation of p38 by XAN, which accounted for the cell cycle arrest at G2 phase, and the downregulation of ERK associated with the proapoptosis activity were also revealed. Further analysis found p53 may be the central role mediated the bioactivities of MAPKs in A549/Taxol cells. Based on these evidences, a conclusion has been deduced that XAN could be a potential agent for MDR NSCLC therapy targeting specifically MAPKs.

## 1. Introduction

*Securidaca inappendiculata* Hassk. (SI), a medicinal plant from genus* Securidaca* mainly yielded in South China, is used to cure fractures and rheumatoid arthritis locally [1]. Our previous research found that xanthones were main bioactive compounds in SI [2–4]. Apart from significant anti-inflammatory and antirheumatic activities, they also exhibited obvious antitumor effects both* in vivo* and* vitro* [5]. More interesting, although they are weaker antirheumatic agents compared with conventional drugs available now, they can efficiently inhibit the proliferation of some types of tumor cells.

Lung cancer is a most commonly occurring tumor. About 1.35 million new cases of lung cancer are diagnosed each year globally which contributes to 12.4% of the total cancer incidence, and it results in 1.18 million deaths yearly which contributes to 17.6% of total deaths from cancer [6]. Several risk factors, such as genetic susceptibility, air pollution, chemical stimulus, and physical inactivity, have been proven with close association with lung cancer, while smoking is widely known as the most important causative factor [7, 8]. With the biggest smoker population, China consumes innumerous cigarettes, which cost over 2.3 trillion Yuan in 2013. As the result of heavy tobacco consumption, China bears a heavy burden of lung cancer [9], and lung cancer especially the major prevalent type non-small cell lung carcinoma (NSCLC) poses a severe threat to public health in China [6]. Similar things happen all over the world.

Although chemotherapy is still an important means of tackling cancers nowadays, low efficiency, severe side effects, and especially multidrug resistance (MDR) become main impediments to achieve a satisfying efficacy in long-term treatments [10]. Available evidences show xanthones have growth inhibitory potency against various tumor cell lines including some MDR types [11–14]. We also found MDR NSCLC lung tumor cell line A549/Taxol was sensitive to xanthones from SI. Documents have already revealed xanthones can repress the proliferation of MDR cell lines via suppression on P-gp and MPR-1 [15–17]; however the underlying mechanism involved in these regulatory actions is still largely unknown. We found xanthones from SI can efficiently modulate MAPKs pathways in several cell lines. Since MAPKs exert great influences on the fate of cells and have a close relationship with MDR, we assume MAPKs are important targets of xanthones in A549/Taxol cells. Xanthones may manipulate proliferation and MDR related genes by selective modulation of MAPKs and decide the fate of cells.

Here we reported the regulatory effects of 1,7-dihydroxy-3,4-dimethoxyxanthone (XAN, a typical xanthone from SI) on MAPKs and the subsequent cellular responses in A549/Taxol cells. This research shed some light on the mechanism of inhibition on MDR tumor cells by xanthones and is beneficial to a better understanding of the acknowledge concerning the association between MAPKs regulation and the selective inhibition on MDR tumor cells.

## 2. Materials and Methods

### 2.1. Reagents

1,7-Dihydroxy-3,4-dimethoxyxanthone (XAN) with purity of 99% was isolated and purified from* Securidaca inappendiculata* Hassk. by our laboratory [5]. 3-(4,5-Dimethylthiazol-2-yl)-2,5-diphenyl-tetrazolium bromide (MTT), BCA protein determination kit, PI cell cycle detection kit, and Annexin V-FITC/PI apoptosis detection kit were purchased from KeyGen Biotech (Jiangsu, China). Rabbit primary antibodies against p-p38, p-JNK, p-ERK, p-AKT, p21, p53, P-gp, bcl-2, cleaved caspase-9 (c-caspase-9), cleaved caspase-3 (c-caspase-3), *β*-actin, and anti-rabbit HPR linked secondary antibody were purchased from Cell Signaling Technology (Beverly, MA, USA). U0126, SB202190 (SB), and SP600125 (SP) (inhibitors of ERK, p38, and JNK, resp.) were supplied by Hanxiang Biotech (Shanghai, China). Roswell Park Memorial Institute 1640 (RPMI 1640), Phosphate Buffered Saline at pH of 7.4 (PBS), and ECL chemiluminescence detection kit were bought from Thermo Scientific (Rockford, IL, USA). Bovine Calf Serum (CS) was supplied by Wisent Inc. (Canada). All other chemicals and reagents used were of analytical grade.

### 2.2. Cell Culture

MDR human lung cancer cell line A549/Taxol and drug sensitive lung cancer cell line A549 were purchased from KeyGen Biotech (Nanjing, China) and cultured in RPMI 1640 supplemented with 10% CS, 100 U/mL penicillin, and 100 *μ*g/mL streptomycin at 37°C in 5% CO_2_ atmosphere. MDR of A549/Taxol cells was maintained by supply of paclitaxel (Taxol, 200 ng/mL) into culture medium, and the agent was removed two weeks prior to experiments. Every 2 days, cells were passaged.

### 2.3. MTT Assay

One day ahead of assay, cells were seeded onto 96-well plates at density of 5 × 10^3^ cells/well and incubated under normal conditions. Varying amounts of XAN were added into medium (final concentrations in medium were 41.7, 20.8, 10.4, 5.2, and 2.6 *μ*M, and only concentration of 20.8 *μ*M was used for time sensitive assay), and same volume of PBS served as negative control. Cells in some wells were cotreated with MAPKs inhibitors as predetermined arrangement. Final concentrations of SB, SP, and U0126 in medium were 25, 25, and 10 *μ*M, respectively. After the incubation, 20 *μ*L MTT solution was added and a further 4 h incubation was carried out. Finally, 150 *μ*L DMSO was used to dissolve MTT formazan salt, and the absorbance was measured on a microplate reader (BIO-RAD Science Co. Ltd., USA) at 490 nm.

### 2.4. Apoptosis and Cell Cycle Analyses

A549/Taxol cells were seeded onto 6-well plates at density of 5 × 10^5^ cells/well. After a 12 h incubation, cells were treated with XAN at the concentration of 5.2 or 20.8 *μ*M (or cotreated with inhibitors as predetermined arrangement) for 24 h. By the end of treatments, cells were divided into two equally for the following flow cytometric analyses.

For the apoptosis analysis purpose, all the cells were collected and washed with PBS. After being stained with Annexin V-FITC/PI detection kit in dark for 10 min, population of apoptotic cells was analyzed according to the manufacturer's instructions.

To analyze cell cycle distribution, the supernatant was discarded. Attached cells were washed by PBS twice and harvested. Obtained cells were fixed in cold 70% ethanol under −20°C for 12 h. After staining with PI kit, cell cycle distribution was analyzed according to the manufacturer's protocols available. All the flow cytometric analysis procedures were performed on a FACS Calibur system (Becton & Dickson, San Jose, CA, USA).

### 2.5. Western-Blot

Cells were seeded onto 6-well plates at the density of 5 × 10^5^ cells/well prior to the assay. Then cells were treated with XAN (or cotreated with inhibitors). After that, cells were collected, washed twice with prechilled PBS, and then lysed. The lysate was centrifuged at 12000 rpm for 10 min under 4°C. The supernatant was collected and boiled for 5 min. Concentration of protein was measured using a BCA protein determination kit. Proteins were separated by SDS-PAGE and then transferred onto polyvinylidene fluoride membranes (PVDF, 0.45 *μ*m, Millipore). After being blocked with BSA (Bovine Serum Albumin), membranes were incubated with the appropriate primary antibodies at 4°C for 12 h followed by an incubation with HPR linked secondary antibody at room temperature for 1 h. Expressions of proteins were detected using a ChemiDoc XRS^+^ system (Bio-Rad Laboratories, Inc., USA).

### 2.6. Statistical Analysis

Results are expressed as mean ± SD. Statistical differences among groups were evaluated by One-Way ANOVA coupled with* post hoc* tests using Excel 2007 software (Microsoft, USA). Differences were considered statistically significant at ^*∗*^ *P* < 0.05, ^*∗∗*^ *P* < 0.01.

## 3. Result

### 3.1. Modulation of MAPKs Involved in Antiproliferation of XAN on A549/Taxol Cells

**Figure 1:**
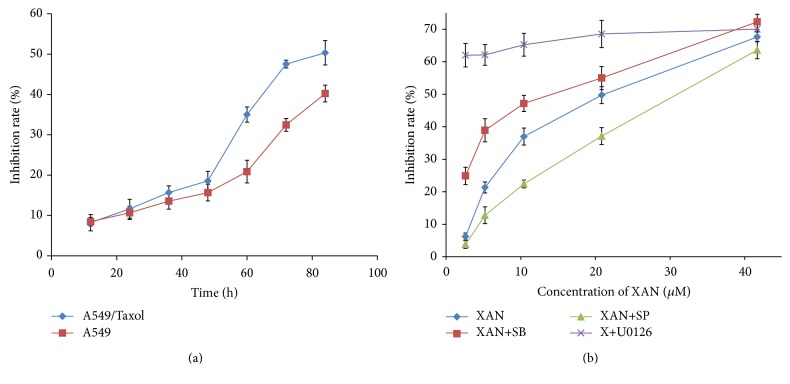
XAN inhibited the proliferation of A549/Taxol (A549) cells* in vitro*. Results were assessed by MTT assay; values are presented as mean ± SD, *n* = 3.

A549/Taxol cells exhibited high sensitivity to XAN suggested by MTT assay. We monitored the viability of A549/Taxol cells continuously up to 84 h. During the first 48 h, the growth inhibition rate increased gradually and rose dramatically in the following 24 h, while no obvious increase was noticed anymore in the rest of the time (Figure 1(a)). Hence, further MTT assay was carried out at 72 h. Under the same experimental procedures, A549 cells were less sensitive to XAN (Figure 1(a)). These results indicated XAN possessed a selectively inhibitory activity on MDR type of tumor cells. Also, it is found that XAN inhibited proliferation of A549/Taxol cells in a concentration dependent manner, and MAPKs inhibitors exerted different effects on the results (Figure 1(b)). U0126 and SB reinforced the inhibitory activity, while SP afforded the contrary outcome. Similar but weaker effects on A549 cells were induced by U0126 and SP compared with the MDR counterpart, while SB attenuated the inhibitory activity of XAN (data not provided). These results suggested that modulation of MAPKs by XAN may be involved in the inhibition of A549/Taxol cells and possibly contribute to the different effects on the two cell lines. Upregulation of JNK and downregulation of ERK and p38 seemed to be preferred for better antiproliferative activity of A549/Taxol cells.

**Figure 2:**
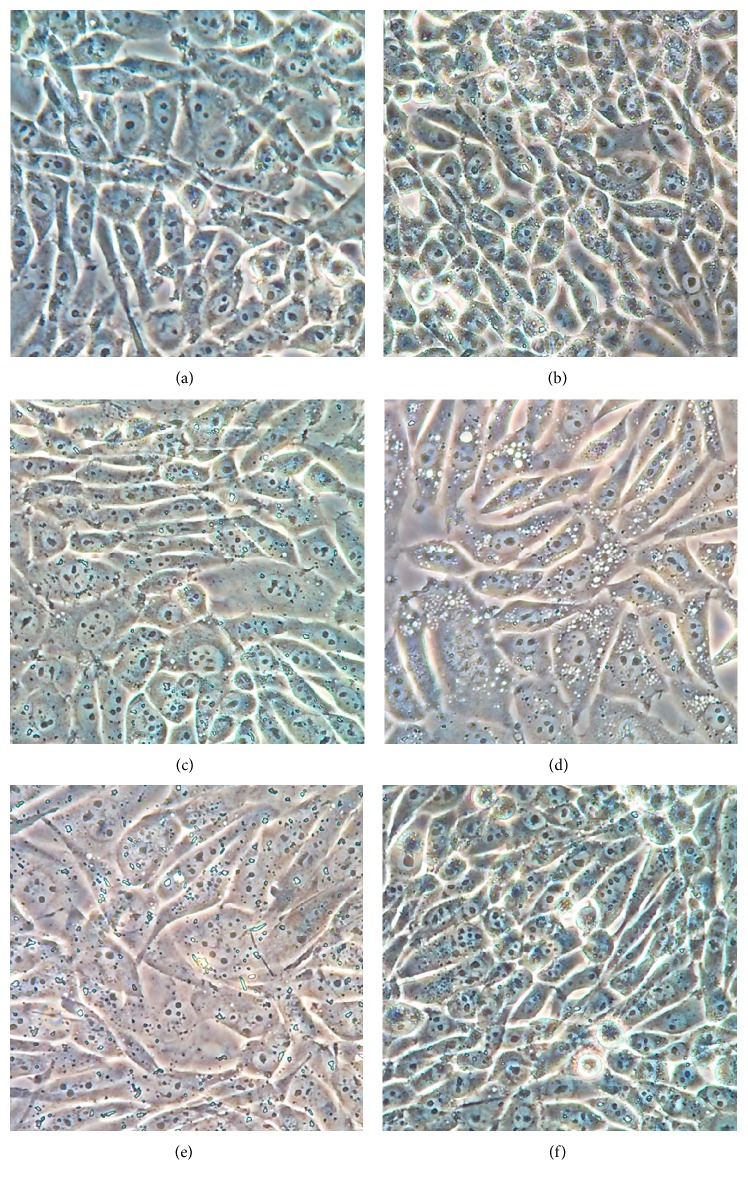
Morphology of A549/Taxol cells. (a) Normal; (b) XAN (5.2 *μ*M); (c) XAN (20.8 *μ*M); (d) XAN (20.8 *μ*M) + SB (25 *μ*M); (e) XAN (20.8 *μ*M) + U0126 (10 *μ*M); (f) XAN (20.8 *μ*M) + SP (25 *μ*M).

Morphology observation provided us with some additional useful clues (Figure 2). XAN induced karyorrhexis of cells at high concentrations. SB restored abnormal changes in nuclei caused by XAN but aggravated swelling and vacuolization of cells. These evidences hinted XAN could inhibit the growth of A549/Taxol cells by activating p38 pathway and revealed the multitarget effects of SB accounting for the cytotoxic effects. Cotreatment with U0126 reinforced the cytotoxicity of XAN indicated by the significant chromolysis, and, contrarily, SP exhibited a protective effect on cells. Taking all these clues together, we think that XAN upregulated p38 and JNK, downregulated ERK pathways, and then exerted the subsequent inhibitory effects on A549/Taxol cells.

### 3.2. Proapoptosis Contributed to Growth Inhibition of XAN on A549/Taxol Cells

**Figure 3:**
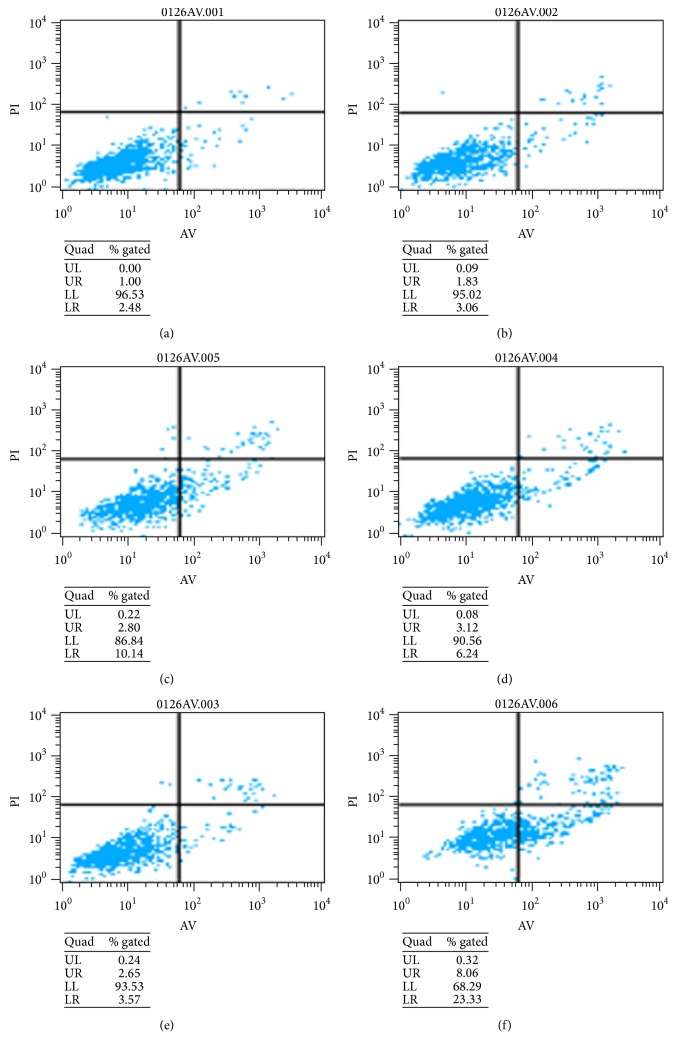
XAN induced apoptosis of A549/Taxol cells, and MAPKs inhibitors exerted divergent effects on the results. (a) Normal; (b) XAN (5.2 *μ*M); (c) XAN (20.8 *μ*M); (d) XAN (20.8 *μ*M) + SB (25 *μ*M); (e) XAN (20.8 *μ*M) + SP (25 *μ*M); (f) XAN (20.8 *μ*M) + U0126 (10 *μ*M).

Morphology observation found signs of apoptosis of cells induced by XAN* in vitro*. To further investigate the proapoptosis effects of XAN, flow cytometry analysis was used. It was found that XAN induced apoptosis in a concentration dependent manner (Figure 3). Although apoptosis increased by the raised concentration of XAN, the apoptotic population (mainly at early stage) was small even at high concentration, and then we came to a conclusion that although XAN could induce mild apoptosis, the weak effect cannot contribute mostly to the inhibition on A549/Taxol cells, and other means should also account for the inhibitory activity. To investigate roles of MAPKs in apoptosis, various inhibitors were added. SP and SB exerted little to the results, while U0126 significantly raised the apoptotic portion. It hinted that apoptosis of A549/Taxol cells was possibly elicited mainly in response to abrogation of ERK by XAN.

### 3.3. XAN Induced G2 Phase Arrest via Upregulation of p38 Signaling

**Figure 4:**
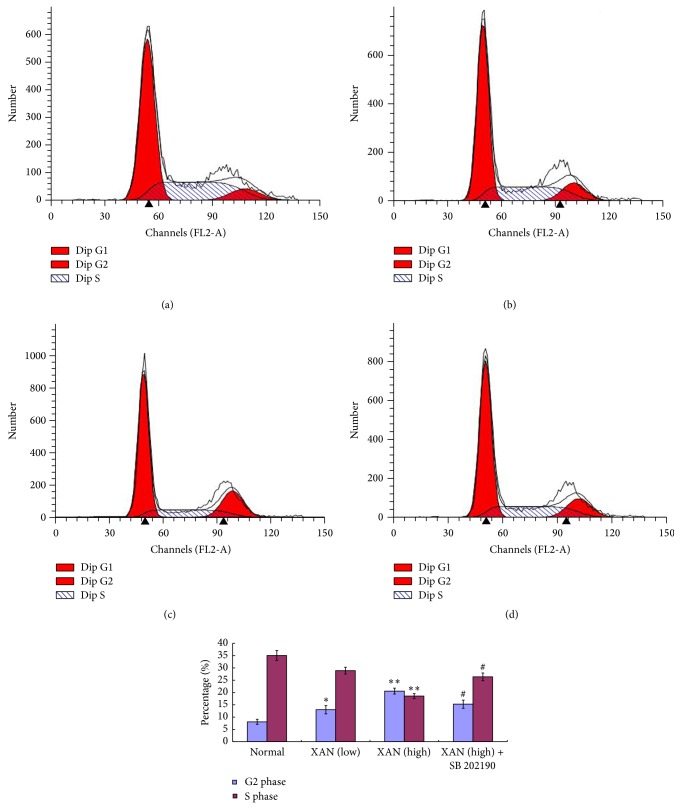
XAN induced cell cycle arrest of A549/Taxol cells at G2 phase, and SB reversed these effects. (a) Normal; (b) XAN (5.2 *μ*M); (c) XAN (20.8 *μ*M); (d) XAN (20.8 *μ*M) + SB (25 *μ*M). ^*∗*^ *P* < 0.05, ^*∗∗*^ *P* < 0.01 compared with normal control; ^#^ *P* < 0.05 compared with XAN (20.8 *μ*M).

Besides apoptosis, cell circle arrest usually contributes great importance to growth inhibition of cells too. Results from MTT assay and morphology observation suggested upregulation of p38 and JNK may link to the inhibitory activity of XAN on A549/Taxol cells, but apoptosis analysis found SB and SP exerted little on the results. Two possible explanations were then deduced: (1) XAN cannot activate the two pathways; (2) activation of the two induced inhibitory effects by means other than proapoptosis. Considering p38 and its downstream signalings play important regulative roles in cell cycle progression, we hypothesized that there existed a possible association between the modulation of p38 by XAN and cell circle arrest in A549/Taxol cells. To test the hypothesis, a cell phase distribution analysis was performed. It is found that XAN arrested cell cycle at G2 phase in a concentration dependent manner. Cotreatment with SB reversed the changes, which strongly supported our claim that XAN activated p38 pathway and elicited subsequent cell cycle arrest (Figure 4). The proposed linkage between them was further tested by Western-Blot assay.

### 3.4. XAN Selectively Modulated MAPKs and Main Downstream Signalings

**Figure 5:**
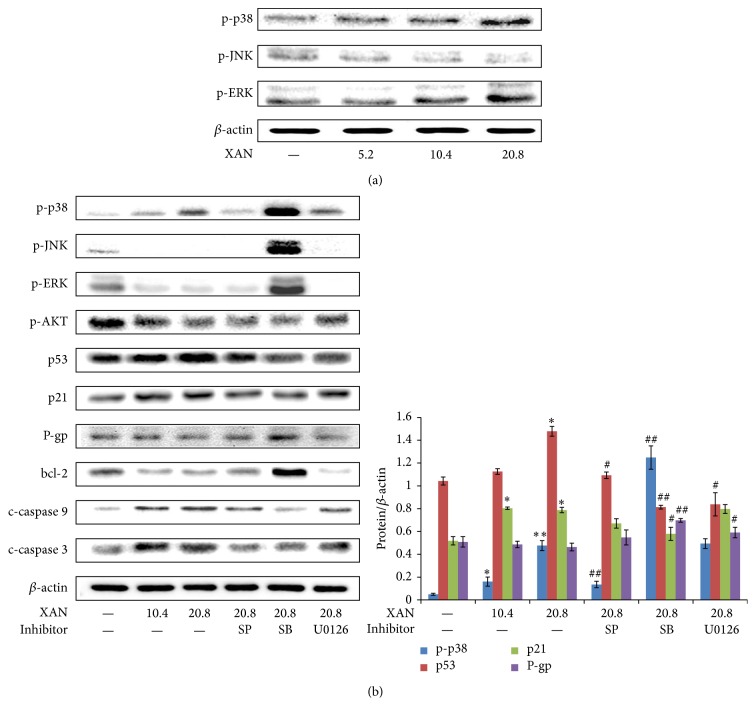
XAN (and inhibitors) modulated MAPKs and main downstream signalings in A549/Taxol cells. (a) 8 h after treatment; (b) 36 h after treatment. Unit of concentration of XAN and inhibitors was *μ*M. ^*∗*^ *P* < 0.05, ^*∗∗*^ *P* < 0.01 compared with normal control; ^#^ *P* < 0.05, ^##^ *P* < 0.01 compared with XAN (20.8 *μ*M).

**Figure 6:**
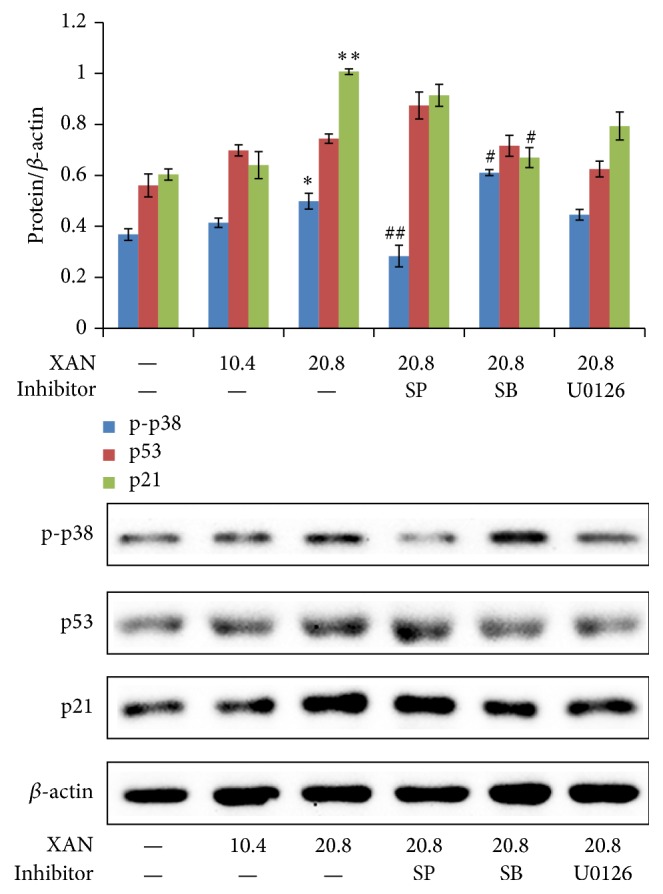
XAN (and inhibitors) modulated MAPKs in A549 cells after the 36 h treatment. Unit of concentration of XAN and inhibitors was *μ*M. ^*∗*^ *P* < 0.05, ^*∗∗*^ *P* < 0.01 compared with normal control; ^#^ *P* < 0.05, ^##^ *P* < 0.01 compared with XAN (20.8 *μ*M).

We firstly investigated expressions of major kinases at two time points (8 h and 36 h after treatment) and found modulation of XAN on MAPKs was time sensitive. At 8 h, XAN elevated p-ERK in a concentration dependent manner, while the result was totally reverted at 36 h (Figure 5). Regulation of p-p38 and p-JNK were not so sensitive to time. Since ERK is an important stress pathway, the transitory activation is common under various stimulus, and XAN mainly exerted inhibitory effects on A549/Taxol cells at the late stage of treatment. Taking all these into consideration, we think changes of kinases at 36 h were more worthwhile and carried out further assay at this time point. Some important relevant proteins were also investigated. Similar to effects on p-p38, XAN obviously upregulated expressions of p53 and p21 (Figure 5(b)), and these effects were proposed as the fundamental reason for cell cycle arrest for their regulatory roles in cell cycle progression. Proapoptosis effect of XAN was confirmed by upregulated c-caspases 9 and 3 and downregulated bcl-2. P-gp was repressed by XAN, suggesting the potential of MDR reversion. Inhibitors cotreatments provided us with important clues to clarifying the roles of MAPKs in the course of inhibition on A549/Taxol cells. SB not only reversed modulation of p53/p21, but also raised levels of p-p38, p-JNK, and p-ERK. It explained the double functions of SB. On one hand, SB inhibited p38 and the downstream p53 signaling and reversed cell cycle arrest; on the other hand, it activated JNK pathway and caused subsequent injuries in cells. U0126 significantly reinforced the effect on bcl-2, suggesting downregulation of ERK was an important means of XAN to induce apoptosis. By a further analysis, the patrol role of p38/p53 was noticed. It was revealed that p53 had a close relationship with the cell cycle arrest, proapoptosis, and MDR reversion activities of XAN suggested by the synchronized changes of p21, bcl-2, c-caspase 9/3, and P-gp. Ablation of p38 by SB suppressed p53 and restored changes mentioned above. Western-Blot analysis also found the regulatory effect of XAN on p38/p53/p21 signaling in A549 cells (Figure 6). The effects were similar but weaker to that in the MDR counterpart, which was possibly caused by the selective inhibition on A549/Taxol cells.

## 4. Discussion

As a global disease, lung cancer poses a great threat on humankind. Although surgical resection is deemed as the best choice for lung cancer patients, most cases are not eligible candidates because they are diagnosed at advanced stage. Chemotherapy provides us with an important alternative, especially for advanced ones. Efficacy of chemotherapy is satisfying during the initial phase of treatment, with a response rate of 20–50% in advanced NSCLC and 60–80% in extensive small cell lung cancer (SCLC). However, final outcome is limited in long term because of acquired MDR with broad cross-resistance to various agents, which leads to lethal recurrence eventually [18]. From this point of view, novel agents sensitive to MDR cancers are in great demand.

Numerous researches found xanthones present notable growth inhibitory activity on MDR tumor cells [19, 20]. These findings raised the question of how xanthones avoid MDR and inhibit the growth of these cells. Although some reports have already revealed the repressive activity of xanthone on MDR related genes/proteins, the mechanism by which xanthone manipulates these signalings and avoids MDR is far away from well understood.

Accumulating insights into the mechanism revealed linkage of reactivation of specific pathways and development of MDR. Among these pathways, MAPKs are well investigated because of their extensive roles in prosurvival and inflammatory responses, and the potential relation to occurrence of MDR. p38 is usually deemed as a tumor suppressor, and its activation in most cases could elicited cell cycle arrest and apoptosis in cells [21, 22]. The negative regulative role of p38 in initiation of MDR was also found [23]. But meanwhile a large volume of reports found that p38 plays a key role in resistance of many chemotherapy drugs [24], and targeted inhibition could enhance the sensitivity of MDR cells to chemotherapy [25]. These findings showed the sophisticated role of p38. We found XAN elevated expression of p-p38 notably in MDR tumor cells and concomitantly inhibited the proliferation of cells. Based on the experimental results, it was believed that upregulation of p38 and the downstream signaling by XAN were closely associated with the cell cycle arrest and subsequent growth inhibition. Similar to our findings, other scholars also found activation of p38 could inhibit the proliferation of MDR lung cancer cells [26]. PI3K/Akt-ERK pathway is usually deemed as a major molecular target for cancer therapy due to its role in promoting MDR [27], and simultaneous inhibition of AKT and ERK often afforded satisfying growth inhibition on MDR cells [28]. For these reasons, suppression on p-AKT and p-ERK of XAN is essential for the proapoptosis activity on A549/Taxol cells which was supported by apoptosis analysis and Western-Blot assay. p-JNK was downregulated by XAN; however it is hard to figure out its contribution to the inhibitory activity on A549/Taxol cells, for JNK is always deemed to have a positive role in promoting apoptosis and overcoming MDR [29], and perhaps it is due to the sophisticated cross-talk among MAPKs pathways [29, 30].

Upon the comprehensive analysis of the obtained results, we found p53 possibly played a central role in these courses. As an important tumor suppressor, p53 not only regulates apoptosis and cell cycle progression, but also modulates mdr1 gene expression. As well known, wild-type p53 can suppress MDR/MRP expression, whereas several common p53 mutants are able to activate them [31, 32]. Clinical evidence has proven that there exists a positive correlation between the mutant p53 and MDR in lung cancer patients [33]. Hence, we assumed XAN could promote the expression of wild-type p53 via regulation on MAPKs and afford the potential of proliferation inhibition and MDR reversion. Meanwhile, xanthone derivatives can inhibit the interaction of p53 with MDM2 [34]. This effect could substantially repress the function of MDR-1 [35]. Based on these clues, it is believed that inhibition on interaction of p53-MDM2 by XAN could contribute to the accumulation of p53 in A549/Taxol cells too.

## 5. Conclusion

As natural occurring compounds, xanthones possess great potential on cancer therapies. Although the efficacy is weaker than conventional chemotherapy agents, their advantages, such as MDR avoiding and low toxicity, are notable. Present study found XAN could efficiently regulate MAPKs and induced subsequent proliferation inhibition in A549/Taxol cells. These findings suggested xanthones could be potential agents for MDR cancers therapy targeting MAPKs and their downstream signalings, such as p53.
